# Experimental investigation and reliability-based optimization of the nut factor in bolted joints considering friction coefficient, surface roughness and material hardness

**DOI:** 10.1371/journal.pone.0351082

**Published:** 2026-06-11

**Authors:** Van Thuy Tran, Huu Loc Nguyen

**Affiliations:** 1 Faculty of Engineering-Technology, Pham Van Dong University, Cam Thanh, Quang Ngai Province, Vietnam; 2 Department of Machine Design, Faculty of Mechanical Engineering, Ho Chi Minh City University of Technology (HCMUT), Ho Chi Minh City, Vietnam; 3 Vietnam National University Ho Chi Minh City, Linh Xuan Ward, Ho Chi Minh City, Vietnam; University of Perugia: Universita degli Studi di Perugia, ITALY

## Abstract

The nut factor is a critical parameter that defines the relationship between tightening torque and axial preload in bolted joints. Accurate estimation of this coefficient is essential to ensure joint integrity and reliability. This study presents a controlled experimental investigation to evaluate the effects of three key assembly parameters including friction coefficient (μ), surface roughness (Ra), and material hardness (HB) on the nut factor (K). A second-order regression model was developed based on experimental data to describe the nonlinear relationship between these variables. Furthermore, a Reliability-Based Design Optimization (RBDO) approach was employed, integrating Monte Carlo simulation and Genetic Algorithm, to determine the optimal combination of μ, Ra, and HB that minimizes the expected nut factor while ensuring that the probability of remaining within the safe range [0.2, 0.3] exceeds 99.9%. The results demonstrate that the RBDO approach yields a more robust and reliable tightening configuration compared to conventional optimization methods. Sensitivity analysis was also conducted to quantify the relative influence of each input variable on the reliability of the nut factor. The proposed methodology demonstrates the potential applicability of probabilistic optimization approaches for improving torque control and reliability performance of bolted assemblies under uncertainty.

## Introduction

Among various mechanical fastening methods, bolted joints maintain a vital role due to their flexibility, ease of assembly and disassembly, and high reliability in axial load transmission. However, in torque-controlled tightening processes, only a fraction of the applied torque is effectively converted into axial preload, while a significant portion is lost to friction at the thread flanks and bearing surfaces. Consequently, the nut factor (K), which characterizes the relationship between tightening torque and resulting preload, becomes a critical parameter that must be accurately estimated to ensure the safety and effectiveness of the joint [1–3].

Numerous studies have investigated the influence of friction on preload variation, demonstrating that even small deviations in the friction coefficient can cause significant discrepancies in the resulting axial force. Surface conditions (dry, lubricated, coated), surface cleanliness, and assembly procedures are all major contributors to frictional variability [4–6]. Friction occurring at the thread interface and under-head surface is considered the dominant mechanism governing torque-to-preload conversion. In addition to friction, surface roughness (Ra) plays an important role as it affects the actual contact area and local friction behavior during tightening. Some studies have shown that smoother surfaces typically exhibit lower friction coefficients, thereby impacting torque efficiency [4,7–10]. However, in many existing investigations, surface roughness is often treated as a secondary or fixed parameter, and its interaction with other influencing variables remains insufficiently explored. Another frequently overlooked yet practically important factor is material hardness (HB) [11–13]. When the clamped components are made from softer materials, plastic deformation at the contact zone increases, altering the actual contact condition and the effective friction. In contrast, harder materials provide better stress distribution and reduce localized indentation. Despite its relevance, material hardness has not been widely included as an independent design variable in many empirical or semi-theoretical models of the nut factor.

In torque-controlled bolted joints, the stability of the nut factor K plays an important role in preload retention and vibration resistance. Excessive variation in K may lead to nonuniform preload generation, increasing the risks of local slip, preload loss, and self-loosening under cyclic vibration conditions [11,14]. Maintaining K within a stable and controlled range can improve tightening consistency and contribute to more reliable joint performance during service. Therefore, optimizing the nut factor is not only important for tightening accuracy but also potentially beneficial for enhancing vibration resistance and long-term joint reliability.

From a modeling perspective, existing empirical or simplified theoretical expressions typically assume idealized conditions, without fully accounting for the combined and interactive effects of multiple influencing factors such as friction, roughness, and material properties. This often leads to significant discrepancies in real-world assemblies, which are inherently subjected to uncertainty due to manufacturing errors, lubrication inconsistency, and material heterogeneity. Compared with advanced AI-based approaches, the RSM framework adopted in the present study provides improved statistical interpretability, efficient modeling under limited experimental datasets, and straightforward integration with reliability analysis and optimization procedures.

In recent years, Design of Experiments (DOE) and Response Surface Methodology (RSM) have been applied to statistically assess the multivariate influences on the torque-preload relationship in bolted joints [15–18]. In parallel, advanced hybrid optimization–prediction frameworks integrating artificial intelligence and metaheuristic algorithms have attracted increasing attention in manufacturing and process optimization studies. For example, Author et al. [19] employed an adaptive neuro-fuzzy inference system (ANFIS) combined with a simulated annealing (SA) algorithm to model and optimize the friction stir welding process of AZ80A Mg alloy joints. Their study demonstrated that intelligent hybrid predictive frameworks can effectively capture nonlinear process behavior and improve optimization performance in complex multi-parameter engineering systems.

However, most existing studies on bolted joint modeling remain limited to deterministic regression-based approaches and do not incorporate reliability analysis techniques to evaluate risks associated with stochastic variability in assembly conditions. Notably, Reliability-Based Design Optimization (RBDO) has emerged as a powerful framework for addressing engineering design problems under uncertainty, particularly in the fields of aerospace, structural mechanics, and fatigue design [20–24]. This approach enables probabilistic constraints to be imposed on design objectives, ensuring that the optimized solution satisfies a specified reliability target. Nevertheless, the application of RBDO in bolted joint design, especially in conjunction with experimentally derived regression models of the nut factor, remains relatively limited in the existing literature [25–29].

In summary, although previous research has considered the individual effects of friction, roughness, or material hardness, there is a clear lack of integrated studies that assess their interactive influence under uncertainty. The combination of controlled experimentation, statistical modeling, and reliability-based optimization represents a promising yet underutilized approach. This research aims to fill that gap by developing a predictive and optimization framework for the nut factor, with direct implications for bolted joint design under real-world operational variability.

Based on this review, it is evident that although multiple factors influencing the nut factor have been studied individually, a comprehensive approach that simultaneously considers friction, surface roughness, and material hardness under stochastic assembly conditions is still lacking. Moreover, there is a scarcity of research that integrates experimental data with modern optimization methods such as RBDO to identify bolted configurations that simultaneously ensure effective load transfer and high reliability.

To address these limitations, this study proposes a comprehensive experimental and computational approach to investigate and optimize the nut factor in bolted joints. The objectives of this research are as follows: (1) To experimentally evaluate the individual and interaction effects of surface roughness, friction coefficient, and material hardness on the nut factor using a second-order regression model; (2) To develop a reliability-based design optimization (RBDO) framework that accounts for uncertainty in assembly conditions using Monte Carlo simulation and Genetic Algorithm; and (3) To analyze the sensitivity of design variables and quantify their impact on reliability performance, thereby providing a robust basis for bolted joint design under uncertainty.

With this integrated approach combining experimental investigation, statistical regression, and reliability-based optimization, the study aims to demonstrate the potential applicability of probabilistic design approaches for bolted joints under complex and uncertain conditions.

## Theoretical background

### Definition and modeling of the nut factor

#### Screw joint theory.

To generate the preload force V in the bolt, a corresponding pre-tightening torque T_V_ must be applied to the bolt body; this torque is determined from the friction torque in the thread pair T_r_ and the friction torque at the contact interface between the nut and the clamping plates T_ms_. Therefore, T_V_ is determined by Equation (1).


$$\begin{array}{c} T_{V}=T_{ms}+T_{r} \end{array}$$ (1)

where, *T*_*ms*_ is the friction moment due to the interface between the nut and clamping plates, *T*_*r*_ is the friction moment from the screw pair.

The friction torque T_ms_ at the contact interface between the nut and the clamping plates is determined according to Equation (2).


$$\begin{array}{c} T_{ms}\text{\textbackslash{}hspace\{0.17em}\;\}=\text{\textbackslash{}hspace\{0.17em}\;\}\frac{Vf}{\text{2}}\left(\frac{D_{\text{0}}+d_{\text{0}}}{\text{2}}\right) \end{array}$$ (2)

Where *f* is the friction coefficient between the nut and clamping plates, *d*_*0*_ is the pitch diameter of the thread hole and *D*_*0*_ is the diameter of the nut.

**Fig 1:**
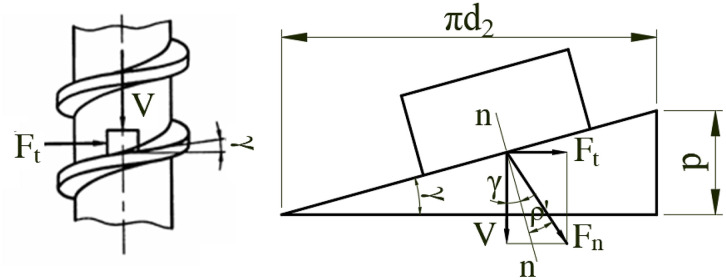
The bolt joint and its loading conditions [1].


The friction torque in the thread T_r_ is determined based on an equivalent inclined-plane model, as illustrated in Fig 1. In this model, the nut is regarded as a sliding body moving on an inclined plane whose inclination angle corresponds to the lead angle of the thread. When friction is taken into account, equilibrium is achieved when the resultant force (F_n_) acting on the nut deviates from the normal direction (n-n) of the inclined plane by an angle equal to the equivalent friction angle ρ′. The forces acting in the model include the axial force V, representing the bolt preload, and the circumferential force F_t_, which is generated by the tightening torque applied to the thread. Based on this equilibrium condition, the relationship between the circumferential force F_t_ and the axial force **V** is determined according to Equation (3).

$$\begin{array}{c} tan\left(\gamma +\rho \prime \right)=\frac{F_{t}}{V} \end{array}$$ (3)

The circumferential force F_t_ is determined by Equation (4).


$$\begin{array}{c} F_{t}=\frac{2T_{r}}{V} \end{array}$$ (4)

Substituting Equation (3) into Equation (4) yields the friction torque T_r_, as expressed in Equation (5).


$$\begin{array}{c} T_{r}=0.5Vd_{\text{2}}\;tan\left(\gamma +\rho \prime \right) \end{array}$$ (5)

Where *f* is the friction coefficient between the nut and clamping plates, *d*_*2*_ is the average diameter of the thread, *γ* is the lead angle and ρ’ is the equivalent friction angle.

Substituting the expressions for *T*_r_ and *T*_*ms*_ into Equation (1) gives:


$$\begin{array}{c} T_{V}=0.5Vd_{\text{2}}\left[\left(\frac{D_{\text{0}}+d_{\text{0}}}{2d_{\text{2}}}\right)f+tan\left(\gamma +\rho \prime \right)\right] \end{array}$$ (6)

#### Nut factor (K).

In bolted joints, the preload force V can be determined indirectly from the tightening moment *T*_*V*_ using the nut factor (K). This relationship is commonly expressed by the following Equation (7).


$$\begin{array}{c} T_{V}=KVd \end{array}$$ (7)

where *d* is the nominal diameter of bolt; *K* is the nut factor that depends on the material, size, surface friction, bolt thread.

The nut factor (K) is an experimental parameter; when compared with the theoretical model of the screw joint given by Equation (6), the corresponding relationship can be established as follows:


$$\begin{array}{c} K=\frac{T_{V}}{Vd}=0.5\left(\frac{d_{\text{2}}}{d}\right)\left[\left(\frac{D_{\text{0}}+d_{\text{0}}}{2d_{\text{2}}}\right)f+tan\left(\gamma +\rho \prime \right)\right] \end{array}$$ (8)

The nut factor (K) represents an empirical parameter that encapsulates the combined effects of various factors governing the relationship between tightening torque and preload force in practical bolted joints. These include friction, torsional and bending effects, elastic deformation of threads, and numerous other known or unknown influences. Due to this complexity, K cannot be precisely determined analytically and is typically evaluated experimentally for each specific application. In practice, a range of K values is often identified to estimate the upper and lower bounds of the preload, thereby facilitating the selection of an appropriate initial tightening torque. The value of K is primarily affected by assembly conditions, lubrication, material properties, and thread surface characteristics. Given the inherent difficulty in accurately measuring the friction coefficients at the nut, bearing surface and thread interface, experimental approaches remain the most reliable method for determining K.

#### Principle of measuring the nut factor.

**Fig 2:**
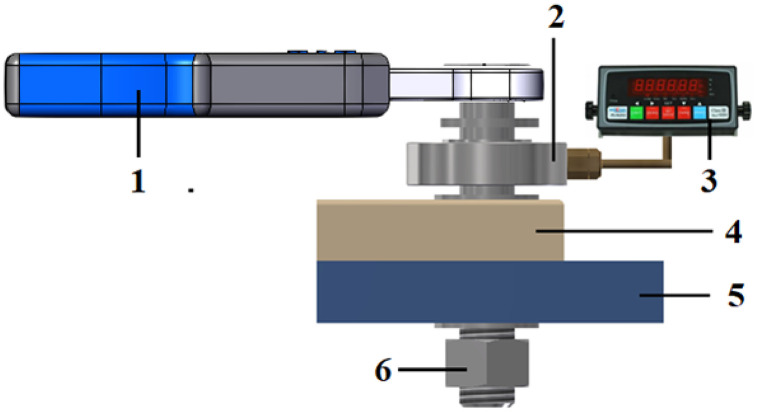
Schematic diagram of the system. Reproduced and modified from Tran et al. [**2**] under the Creative Commons Attribution License (CC BY 4.0).

The measuring system is designed to determine the nut factor (K) in threaded joints by measuring both preload force V and tightening moment T_V_. The schematic diagram of the device operation is illustrated in Fig 2.

*1. Digital torque wrench measuring tightening moment; 2. Loadcell measuring bolt tightening force; 3. Bolt tightening force display meter; 4,5. Clamping plates; 6. Bolted joints M14.*

Measurement Principle of nut factor: The experimental specimen consists of two metal clamping plates connected by an M14 bolt through a circular hole. During testing, the torque wrench (1) applies a controlled tightening moment T to the M14 bolt (6), while the load cell (2) integrated between the clamping plates (4 and 5) records the resulting preload force V and the force data is transmitted to the display meter (3), which provides real-time monitoring. Signal outputs are transmitted to a data acquisition system and displayed via a force meter. By synchronizing the torque and preload measurements, the nut factor can be accurately determined under different surface and material conditions.

### Reliability-Based Design Optimization (RBDO) framework

#### RBDO problem formulation.

The reliability-based optimization problem is formulated as follows [26]:

Objective function: minf(X)


$$\begin{array}{c} \text{Subject to:}\begin{array}{cc} & \left\{ \begin{array}{c} @c@{\text{h}}_{\text{k}}{\text{(X, m}}_{\text{p}}\text{)}\leq \text{0}\\ \overset{\text{l}}{\underset{\text{i}}{\text{X}}}\;\leq {\text{X}}_{\text{i}}\;\leq \overset{\text{n}}{\underset{\text{i}}{\text{X}}}\\ \text{P}\left({\text{g}}_{\text{j}}\left(X,p\right)\geq 0\right)\geq \overset{*}{\underset{j}{R}} \end{array}\right. \end{array}\text{\textbackslash{}hspace\{0.33em}\;\}\begin{array}{c} k=\begin{array}{cccc} 1, & 2, & ... & n_{h} \end{array}\\ i=\begin{array}{cccc} 1, & 2, & ... & n \end{array}\\ j=\begin{array}{cccc} 1, & 2, & ... & n_{g} \end{array} \end{array} \end{array}$$ (9)

where *X* ={*X*_*1*_*, X*_*2*_*, … X*_*n*_} is the design variable (deterministic or random), **X** is the design variable vectors (including deterministic and random), **p** are random parameter vectors (mean value is m_**p**_), f(**X**) is the objective function, g_j_(**X, p**) is the limit state function, n are the numbers of design variables, n_g_ are the numbers of probability constraints, R_j_^*^ are the desired reliability, X_i_^l^, X_i_^n^ are the lower and upper limits of the design variable.

The Monte Carlo simulation method combined with the Genetic Algorithm (GA) is applied to solve the reliability-based design optimization (RBDO) problem for the nut factor K. This approach allows direct handling of the randomness of input variables as well as probabilistic constraints, without requiring derivative information.

#### The Monte Carlo simulation method.

**Fig 3:**
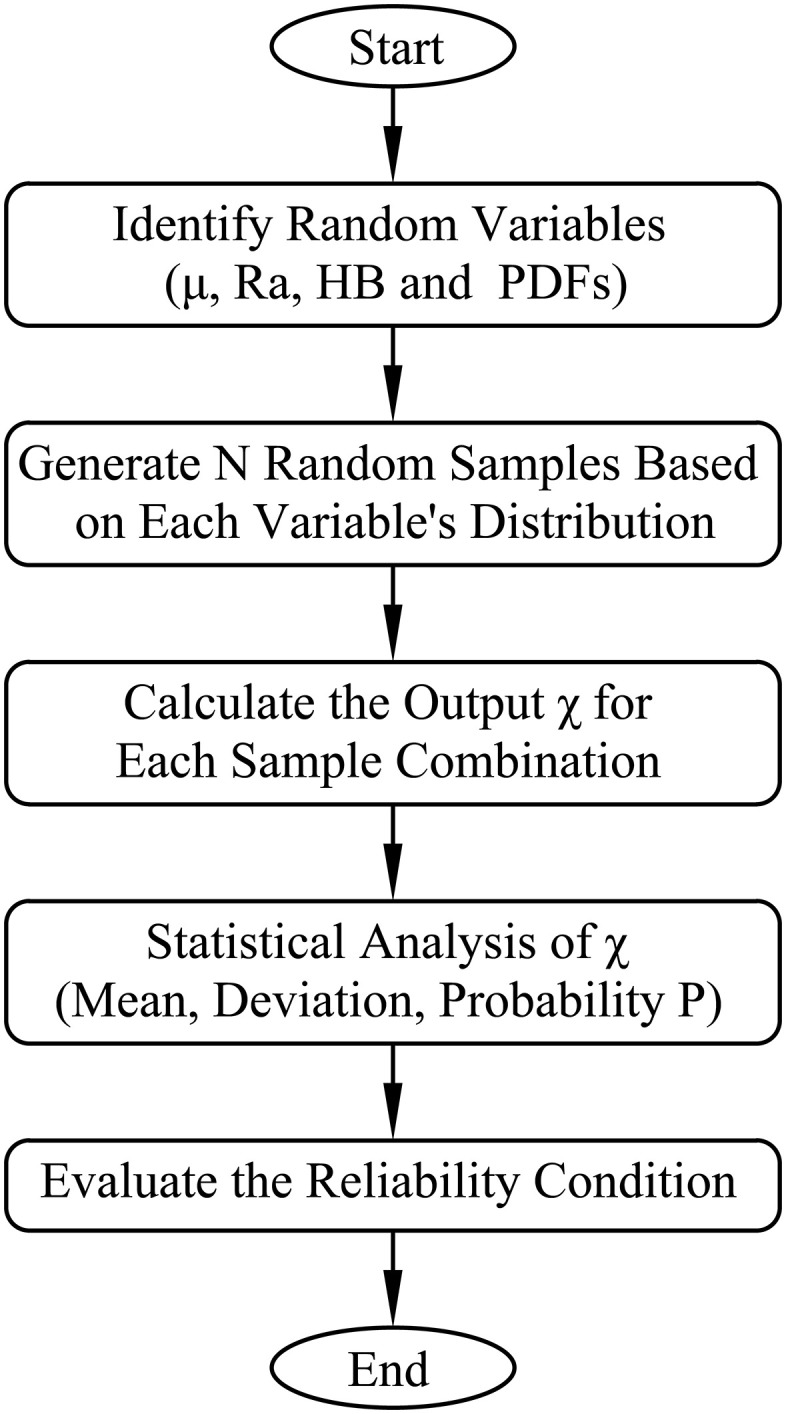
Monte Carlo simulation procedure.

The Monte Carlo Simulation (MCS) is a probability-based simulation technique widely used to analyze systems with uncertain input parameters. In this study, MCS is applied to simulate the distribution of the nut factor K in threaded joints, where the input parameters such as the Combined friction coefficient μ, surface roughness Ra, and material hardness HB are treated as random variables due to manufacturing deviations and assembly conditions. The Monte Carlo simulation procedure is illustrated in Fig 3.

To evaluate the probabilistic constraint, for each individual X=(μ,Ra,HB) generated by the Genetic Algorithm (GA), the random variables are simulated according to a normal distribution:


$$\begin{array}{c} \mu \;\sim \text{ N}\left(\mu ,\sigma_{\mu }\right),\text{ Ra }\sim \text{ N}(\text{Ra},\sigma_{\text{Ra}}),\text{ HB }\sim \text{ N}(\text{HB},\sigma_{\text{HB}}) \end{array}$$

Then, a Monte Carlo simulation with a sufficiently large number of samples is performed to generate a set of K values, from which the probability is estimated.

#### Genetic Algorithm (GA).

**Fig 4:**
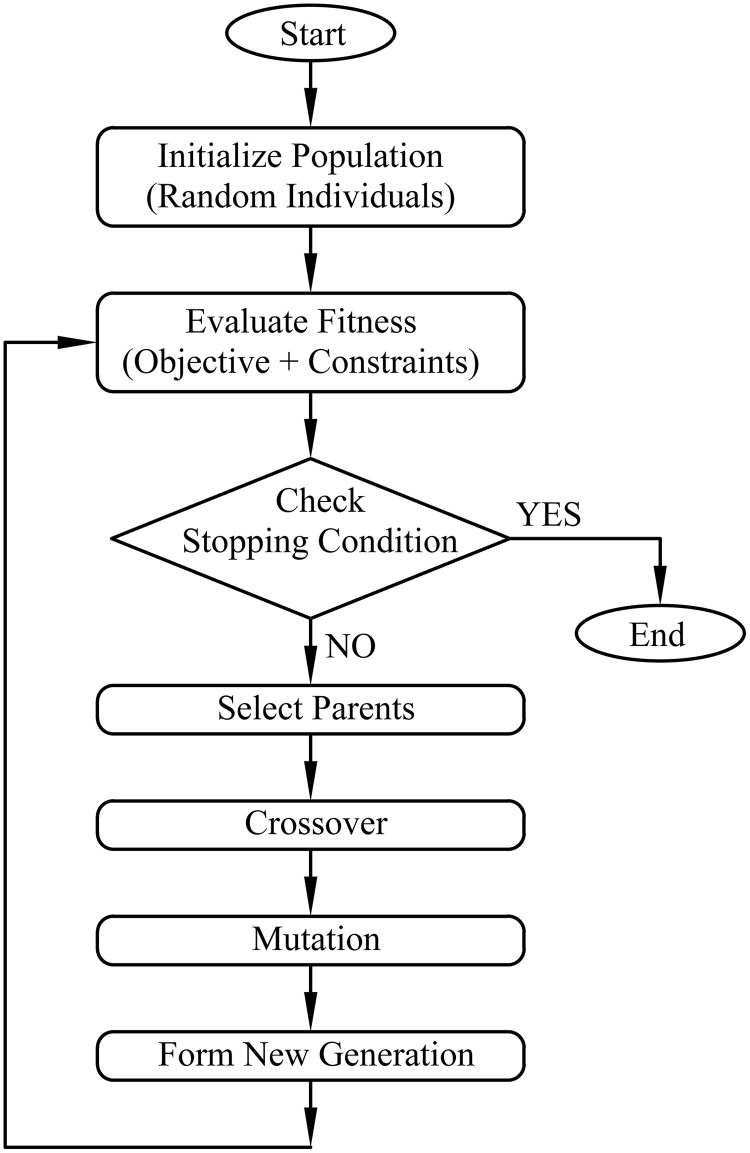
The GA-based optimization procedure.

The Genetic Algorithm (GA) is a heuristic optimization method inspired by the natural evolutionary process, originally proposed by John Holland in the 1970s. GA is particularly effective in solving nonlinear, non-differentiable optimization problems, especially those with multiple local optima or undefined objective functions. Unlike traditional gradient-based optimization algorithms, GA does not require derivative information but relies on a global search mechanism through selection, crossover, and mutation operations applied to a population of candidate solutions. In this study, GA is employed to find the optimal values of the design variable set X. The GA-based optimization procedure is illustrated in Fig 4.

**Fig 5:**
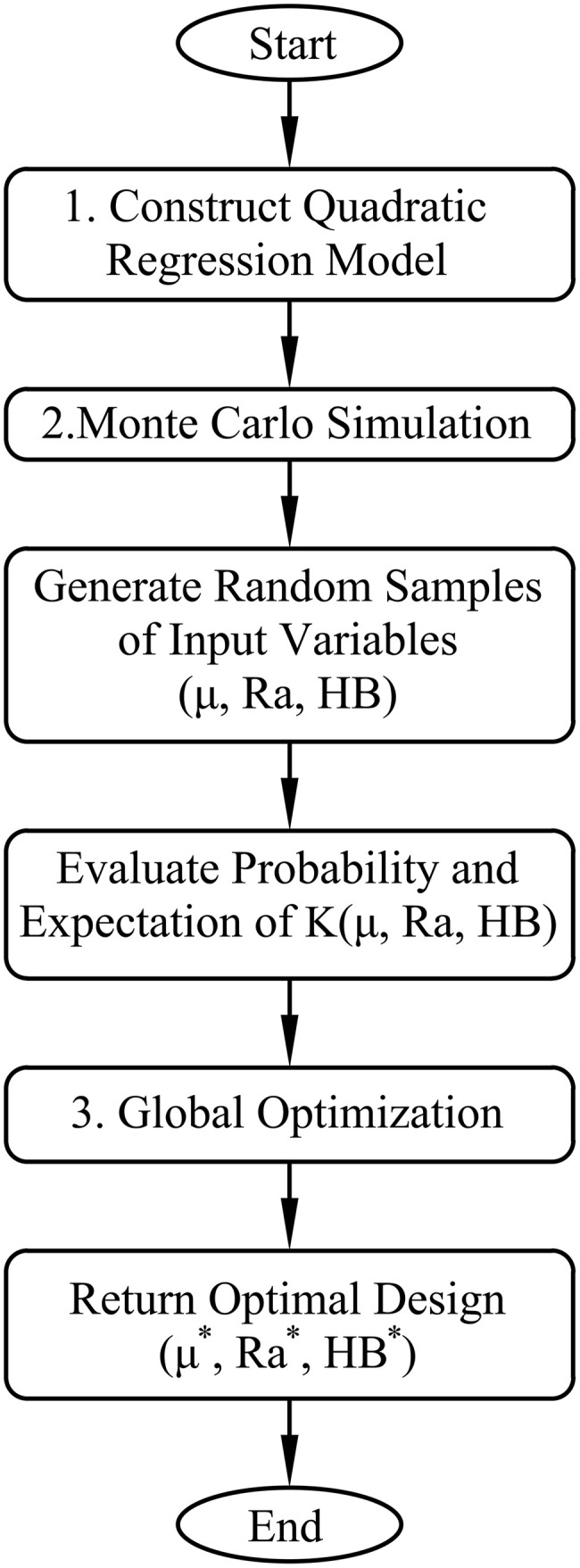
The overall RBDO procedure.

In general, the overall RBDO procedure is illustrated in Fig 5. First, a second-order regression model is constructed to describe the relationship between the nut factor K and the random input variables, including the composite friction coefficient μ, surface roughness Ra, and material hardness HB. Then, the Monte Carlo Simulation (MCS) method is applied to evaluate the uncertainty of these variables by generating a set of random samples according to specified probability distributions.

Next, the regression model is used to compute important statistical indicators such as the expected value of K and the probability that K falls within a predefined safe range. Based on this, a global optimization algorithm is implemented to determine the optimal set of design parameters (μ*,Ra* and HB*) that not only minimizes the objective function but also satisfies the probabilistic constraint at the required reliability level. The final result is an optimal design configuration that accounts for the influence of random factors in the assembly process of threaded joints.

## Experimental methodology

### Experimental setup

**Fig 6:**
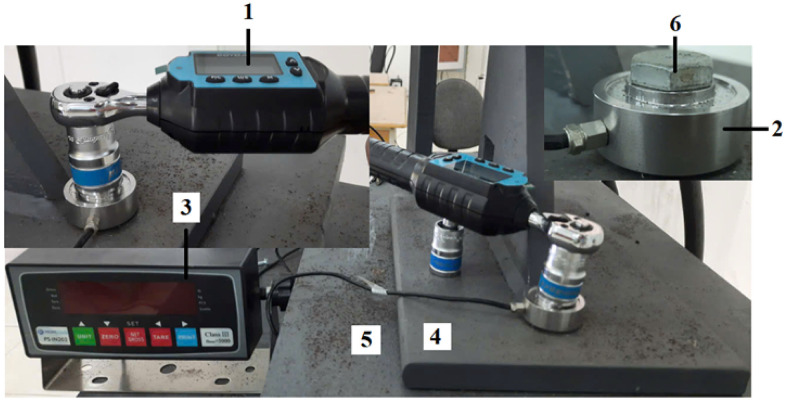
Experimental Setup. Reproduced and modified from Tran et al. [**2**] under the Creative Commons Attribution License (CC BY 4.0).

The experimental setup for determining the nut factor (K) is shown in Fig 6.

*1. Digital torque wrench measuring tightening moment; 2. Loadcell measuring tightening force; 3. Bolt tightening force display meter; 4,5. Clamping plates; 6. Bolt M14.*

The test specimen consists of two metal plates connected by an M14 bolt through a circular hole, to which a tightening torque (T_V_) is applied. Two measurement devices were employed: a digital torque wrench (SK11 SDT3–060, torque range 3–60 N·m, ± 2.5% CW accuracy) to control the applied tightening moment. The torque wrench supports multiple units (N·m, kgf·cm, lbf·in, lbf·ft) and provides peak and tracking modes with audio and visual indicators. A hollow cylindrical loadcell (Donut-type, model FBDA, Forsentek, range 0–20 kN) was placed coaxially with the bolt to measure the corresponding tightening force under external loading. The sensors were factory-calibrated prior to testing. The loadcell specifications include ±0.05% full-scale accuracy, ± 0.02% repeatability, and a linearity error of ±0.1%, ensuring high precision and stability during testing.

To evaluate measurement repeatability, each experimental condition in the Box–Behnken design was repeated three times under identical tightening conditions. The measured preload values showed only minor deviations, confirming the stability and consistency of the torque–tension measurement system. Based on the sensor specifications and repeated measurements, the overall uncertainty bounds of the torque and preload measurements were estimated to remain within ±3%, which was considered acceptable for regression modeling and reliability analysis.

All tightening experiments were conducted under stable laboratory environmental conditions at an ambient temperature of approximately (25 ± 2)^°^C and relative humidity of approximately 60–70%. Environmental conditions were maintained consistently throughout the experimental campaign to minimize their influence on friction behavior and preload variability.

#### Design of Experiment (DoE).

The three input factors including friction coefficient (μ), surface roughness (Ra), and material hardness (HB) are selected to study their effects on the nut factor (K) in threaded joints. These factors significantly influence load distribution and represent key assembly characteristics in mechanical systems.

Although additional parameters such as thread geometry, lubrication condition, tightening speed, washer configuration, and coating state may also influence the nut factor, the present study intentionally focuses on the friction coefficient (μ), surface roughness (Ra), and material hardness (HB). These variables were selected because they directly govern the contact mechanics and torque-to-preload conversion behavior, while remaining experimentally controllable and statistically manageable within the proposed Box–Behnken and RBDO framework. Furthermore, several secondary assembly effects, particularly lubrication and surface treatment conditions, are partially reflected through the effective friction coefficient and surface characteristics. The incorporation of additional assembly parameters will be considered in future multi-factor reliability studies.

The combined friction coefficient (μ) governs the conversion of tightening torque into clamping preload in bolted joints. Due to frictional torque losses at the thread and bearing interfaces, only a portion of the applied torque contributes to preload generation, which directly affects the nut factor (K). According to ISO 16047, typical friction coefficient values for threaded fasteners generally range from 0.1 to 0.3. A value of (μ = 0.1) is commonly associated with well-lubricated contact conditions, whereas (μ = 0.3) corresponds to dry, rough, or poorly lubricated surfaces. These representative levels were selected to cover typical engineering friction conditions for experimental modeling.

In this study, the friction coefficient (μ) was treated as an effective tribological parameter representing different practical contact conditions commonly encountered in bolted joints. The selected (μ) values were physically associated with controlled variations in surface condition and lubrication state rather than representing an independently imposed intrinsic material property.

Surface roughness (Ra) affects real contact area and thus friction during tightening. Rougher surfaces decrease the contact area, increase friction, and lead to variations in clamping force, thereby changing K. Two levels were used: Ra = 1.6 μm (smooth, ground surfaces) and Ra = 6.3 μm (rough, worn surfaces). Surface measurements were made with a Mitutoyo SJ-210 tester (ISO 1997), using a diamond stylus and averaging three measurements per specimen for accuracy. Although (Ra) was selected as the representative roughness parameter in this study due to its widespread industrial use and standardized characterization capability, additional surface descriptors such as (Rz), skewness, and three-dimensional topographical parameters may further improve the understanding of tribological contact behavior and should be investigated in future studies.

In the present study, the surface roughness parameter (Ra) was controlled uniformly for both the thread contact surfaces and the bearing contact surfaces involved in the tightening process. Although the individual friction contributions of these interfaces may differ, the current work focused on evaluating the overall influence of representative surface condition variations on the nut factor

Material hardness (HB) of clamped plates also impacts K. Softer materials deform more under load, increasing bolt load and K, while harder materials distribute load better and reduce K. Two levels were chosen: HB ≈ 120 (mild steel) and HB ≈ 250 (quenched steel). Hardness was measured using a Wilson Rockwell tester (Model 574), compliant with ASTM E18 and ISO 6508 standards. The device supports multiple Rockwell scales and digital output for analysis.

The selected hardness levels were achieved using representative steel conditions with different mechanical hardness characteristics. Although hardness variation may also introduce coupled metallurgical and tribological effects, all specimens were tested under identical geometric and tightening conditions to minimize confounding influences.

Accordingly, when conducting experiments using Box-Behnken design (BBD) with a second-order response surface model [18,30], the value ranges of the three input factors are summarized in Table 1.

**Table 1:** Input factors and their levels.

Variables or parameter	Factor symbol		interval of variation	Variable range of input factors		
	Actual factor	Code factor		level (−1)	level (0)	level (+1)
Friction coefficient, μ	μ	x_1_	0.1	0.1	0.2	0.3
Surface roughness Ra, μm	Ra	x_2_	2.35	1.6	3.95	6.3
Material hardness, HB	HB	x_3_	65	120	185	250

The average nut factor ($\overset{―}{K}$) obtained from the 15 experimental runs is summarized in Table 2. Each experimental condition was repeated three times under identical tightening conditions, and the reported values correspond to the averaged measurements. The applied tightening torque on the bolt was maintained at T = 30 kN ⋅ mm.

**Table 2:** Experimental results of the nut factor measurements under a tightening torque of T = 30 kN⋅ mm.

N_0_	Factor symbol			Friction coefficient	Surface roughness	Material hardness	Nut factor				Standard deviation	Coefficient of variation
	x_1_	x_2_	x_3_	μ	Ra, (μm)	(HB)	*K*_*1*_	*K*_*2*_	*K*_*3*_	$\overset{―}{K}$	SD	CV(%)
1	−1	−1	0	0.1	1.6	185	0.1193	0.1233	0.1228	**0.1218**	0.0022	1.79
2	1	−1	0	0.3	1.6	185	0.1388	0.1418	0.1418	**0.1408**	0.0017	1.23
3	−1	1	0	0.1	6.3	185	0.1593	0.1568	0.1588	**0.1583**	0.0013	0.84
4	1	1	0	0.3	6.3	185	0.1613	0.1618	0.1578	**0.1603**	0.0022	1.36
5	−1	0	−1	0.1	3.95	120	0.292	0.296	0.2925	**0.2935**	0.0022	0.74
6	1	0	−1	0.3	3.95	120	0.2574	0.2614	0.2609	**0.2599**	0.0022	0.84
7	−1	0	1	0.1	3.95	250	0.1251	0.1285	0.1283	**0.1273**	0.0019	1.50
8	1	0	1	0.3	3.95	250	0.1803	0.1831	0.1829	**0.1821**	0.0016	0.86
9	0	−1	−1	0.2	1.6	120	0.264	0.261	0.261	**0.262**	0.0017	0.66
10	0	1	−1	0.2	6.3	120	0.3009	0.2982	0.2985	**0.2992**	0.0015	0.50
11	0	−1	1	0.2	1.6	250	0.1508	0.1481	0.1484	**0.1491**	0.0015	0.99
12	0	1	1	0.2	6.3	250	0.1665	0.169	0.1685	**0.168**	0.0013	0.79
13	0	0	0	0.2	3.95	185	0.2050	0.207	0.2045	**0.2055**	0.0013	0.64
14	0	0	0	0.2	3.95	185	0.2017	0.2047	0.2047	**0.2037**	0.0017	0.85
15	0	0	0	0.2	3.95	185	0.2050	0.2073	0.2066	**0.2063**	0.0012	0.57

The repeated measurements exhibited only minor statistical scatter, with standard deviation (SD) values ranging from 0.0012 to 0.0022 and coefficient of variation (CV) values ranging from 0.49% to 1.79%, with an average CV below 1%. The low SD and CV values confirm the excellent repeatability, consistency, and stability of the torque–tension measurement procedure.

## Regression modeling and analysis

### Regression equation and coefficients

Based on the Box–Behnken experimental model and using Minitab software, the coefficients of the regression equation were determined as follows:


$$\begin{array}{c} \begin{array}{c} K=0.5692+0.7765\;\mu +0.0554\;Ra-0.0052\;HB-0.018\;\mu Ra+0.0034\;\mu HB+\\ -0.00003\;RaHB-3.2044\;\mu^{\text{2}}-0.0051\;Ra^{\text{2}}+0.00001\;HB^{\text{2}} \end{array} \end{array}$$ (10)

After obtaining the regression Equation (10), the next step is to evaluate the statistical significance of the regression coefficients and testing lack of fit in regression model.

### Statistical validation of the model

#### Testing regression coefficients.

The significance of the regression coefficients is evaluated using the t-test for each coefficient, with a predetermined significance level (commonly 5%). Coefficients with P-values less than 0.05 are considered to have a significant effect on the output variable (K), indicating statistical significance. Conversely, coefficients with P-values greater than 0.05 may be considered for removal if they do not substantially affect the model’s accuracy.

The significance of the regression coefficients was assessed using Minitab software. Statistical analysis of the regression model showed that the P-values of all coefficients, including linear terms, quadratic terms, and interaction terms, were smaller than 0.05. This indicates that all investigated factors are statistically significant at the 95% confidence level.

To further evaluate the robustness and statistical reliability of the developed regression model, the confidence intervals of the regression coefficients were also analyzed and are summarized in Table 3. The table includes the estimated coefficients, standard errors, t-statistics, P-values, and the corresponding 95% confidence intervals for all terms of the response surface model. The relatively small standard errors and narrow confidence intervals indicate that the estimated regression coefficients are statistically stable and robust within the investigated parameter range.

**Table 3:** Statistical significance and confidence interval analysis of regression model coefficients.

Term	Coef	SE Coef	95% CI	T	P	VIF
Constant	0.5692	0.006071	(0.55359, 0.58481)	93.756	<0.001	
μ	0.7765	0.02545	(0.71108, 0.84192)	30.292	<0.001	1.00
Ra	0.0554	0.001007	(0.052811, 0.057989)	54.773	<0.001	1.00
HB	−0.0052	0.000048	(−0.0053234, −0.0050766)	−109.176	<0.001	1.00
μ*μ	−3.2044	0.049394	(−3.3314, −3.0774)	−64.018	<0.001	1.01
Ra*Ra	−0.0051	0.000089	(−0.0053288, −0.0048712)	−56.173	<0.001	1.01
HB*HB	0.00001	0.000000	(9.743x10^-6^, 1.026x10^-5^)	85.343	<0.001	1.01
μ*Ra	−0.018	0.002019	(−0.02319, −0.01281)	−10.009	0.003	1.00
μ*HB	0.0034	0.000073	(0.0032123, 0.0035877)	46.569	<0.001	1.00
Ra*HB	−0.00003	0.000003	(−3.771 x10^-5^, −2.229 x10^-5^)	−9.64	0.005	1.00

To further assess potential multicollinearity among the regression terms, the variance inflation factor (VIF) values were evaluated and are included in Table 3. The obtained VIF values were approximately equal to 1 for all linear, quadratic, and interaction terms, indicating negligible multicollinearity within the developed response surface model. These results confirm that the estimated regression coefficients and their statistical significance were not artificially inflated by correlation among predictor variables.

#### Testing the lack of fit in regression.

For testing the lack of fit in regression, the analysis of variance (ANOVA) for signiﬁcance of regression is shown in Table 4

**Table 4:** Analysis of Variance for Signiﬁcance of Regression.

Source	DF	Seq SS	Adj SS	Adj MS	F	P
**Regression**	9	0.047237	0.047237	0.005249	3792.73	0
**Residual Error**	5	0.000007	0.000007	0.000001		
**Lack-of-Fit**	3	0.000006	0.000006	0.000002	2.92	0.266
**Pure Error**	2	0.000001	0.000001	0.000001		
**Total**	14	0.047243				
**R**^**2**^ **= 99.95%; R**^**2**^**(pred) = 99.92%; R**^**2**^**(adj) = 99.93%**						

The analysis of variance (ANOVA) results in Table 4 indicate that the regression model constructed for predicting the nut factor ((K)) is statistically significant. The P-value for the regression source is 0.000, which is less than 0.05, confirming that the overall model is highly significant at the 95% confidence level. This implies that the model effectively explains the variability in the response variable based on the selected input factors and their interactions.

The lack-of-fit P-value is 0.266, which is greater than 0.05. This suggests that there is no statistically significant lack of fit in the model, indicating that the regression equation adequately represents the experimental data and that no major model terms are missing.

The pure error component was estimated from the replicated center-point experiments, resulting in a pure error degree of freedom (DF) of 2 and a pure error mean square (Adj MS) of 10^−6^. The relatively small pure error value together with the non-significant lack-of-fit result further supports the adequacy and statistical reliability of the developed response surface model.

Furthermore, the model shows exceptionally high goodness-of-fit metrics: R-squared (R²) = 99.95%, indicating that 99.95% of the variability in the nut factor (K) can be explained by the regression model. The predicted coefficient of determination R^2^(pred) = 99.92% and adjusted coefficient of determination R^2^(adj) = 99.93% are both very close to the overall R^2^ value. The close agreement among R^2^, R^2^(adj) = 99.93%, and R^2^(pred) indicates that the developed regression model not only exhibits excellent goodness-of-fit but also maintains strong predictive consistency within the investigated parameter space. In particular, the small difference between R^2^ and R^2^(pred) suggests that the model does not exhibit significant overfitting under the selected experimental conditions.

The high (R^2^) value obtained in this study should be interpreted within the context of the controlled experimental design and limited parameter space investigated. The developed response surface model is intended as a local approximation for the selected operating conditions rather than as a universal tribological prediction model

In summary, the regression model constructed based on the Box-Wilson experimental design is statistically valid, highly explanatory, and predictive. It effectively quantifies the influence of the selected factors on the nut factor K and can serve as a reliable basis for further optimization or design studies involving bolted joints.

#### Residual analysis in the regression model.

**Fig 7:**
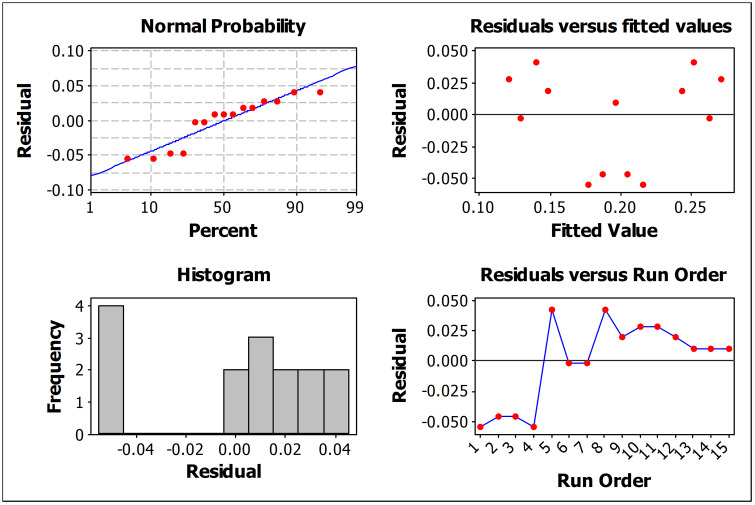
Diagnostic plots for evaluating the residual behavior of the regression model.

To assess the adequacy and reliability of the regression model predicting the load transfer coefficient (K) in bolted joints, residual plots were thoroughly analyzed. These include the normal probability plot, the residuals versus fitted values plot, the residual histogram, and the residuals versus observation order plot, as shown in Fig 7.

The residual plots presented in Fig 7 provide diagnostic validation for the adequacy and assumptions of the regression model for predicting the nut factor K.

***Normal Probability Plot:*** The residuals generally follow a straight-line pattern, indicating that they are approximately normally distributed. This supports the normality assumption required for the validity of ANOVA and regression inference.

***Residuals versus Fitted Values:*** The residuals are randomly dispersed around the zero reference line without any obvious funnel-shaped pattern or systematic structure, suggesting that the variance of errors remains reasonably constant across the range of fitted values.

***Histogram Plot:*** The histogram exhibits an approximately symmetric distribution of residuals. Although the number of observations is relatively limited, no pronounced skewness or abnormal residual concentration is observed, providing additional support for the assumption of approximate normality.

***Residuals versus Run Order***: No significant trend or systematic variation is observed with respect to the observation sequence, indicating the absence of apparent order-dependent bias in the experimental data and supporting the assumption of residual independence.

Overall, the residual diagnostic plots indicate that the assumptions underlying the regression analysis are reasonably satisfied. The residuals exhibit approximately normal behavior, random dispersion, and no significant systematic trend, supporting the adequacy and statistical reliability of the developed response surface model within the investigated parameter range.

#### Analysis of the main and interaction effects of input factors.

**Fig 8:**
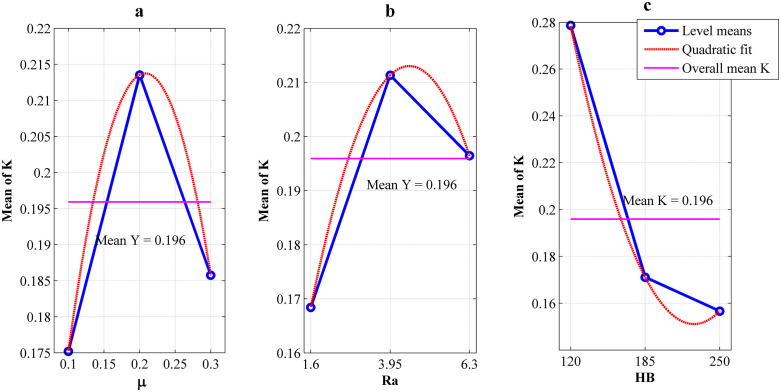
Main Effects Plot. **(a)** Combined friction coefficient; **(b)** Surface roughness; **(c)** Material hardness.

To evaluate the influence of input factors on the output variable in the experimental process, main effects plots were employed. The main effects plot reveals the influence of three independent variables including friction coefficient (μ), surface roughness (Ra), and material hardness (HB) on the average nut factor K, as shown in Fig 8.

***Friction Coefficient (μ):*** The mean value of K increases with μ from 0.1 to 0.2, reaching a peak, and then slightly decreases at μ = 0.3. This suggests a non-linear relationship where an optimal friction level contributes to a higher nut factor before it declines, possibly due to energy loss through excessive friction.

***Surface Roughness (Ra):*** K increases from Ra = 1.60 µm to 3.95 µm and then slightly decreases at Ra = 6.30 µm, indicating that moderate roughness provides more stable contact conditions and thus higher torque efficiency.

***Material Hardness (HB):*** K significantly decreases as HB increases from 120 to 250. This trend suggests that softer materials (lower HB) may lead to higher contact compliance, increasing friction-induced torque and hence a larger nut factor.

In summary, the analysis demonstrates that all three investigated factors significantly affect the nut coefficient, with clear nonlinear or inverse trends. This underscores the importance of rational design parameter selection to optimize the performance of the system.

### Response surface and contour analysis

Following the development and validation of the regression model for predicting the nut factor (K), the model was employed to investigate the combined effects of input parameters. Contour and surface plots were used to visualize parameter interactions and identify regions associated with optimal K values. Unlike single-factor analysis, surface plots reveal optimal operating zones and suitable parameter combinations, enhancing the design reliability of bolted joints.

**Fig 9:**
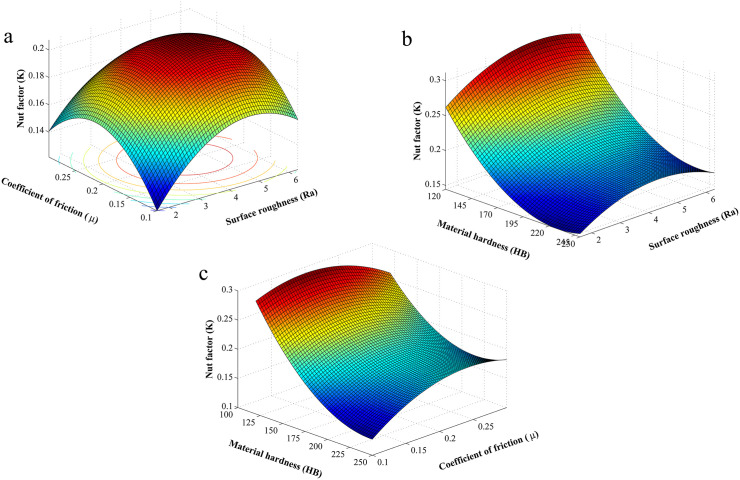
The effects of input factors on the nut factor. **(a)** Effect of μ and Ra on the K; **(b)** Effect of HB and Ra on the K; **(c)** Effect of HB and μ on the **K.**

Fig 9 presents the response surface plots showing the interaction effects of the coefficient of friction (μ), surface roughness (Ra), and material hardness (HB) on the nut factor (K).

***Effect of (μ) and (Ra) on (K):*** Fig 9.a shows that (K) increases with (μ) and (Ra) up to an optimal region (μ ≈ 0.2, Ra ≈ 4 µm), after which it gradually decreases. This behavior can be physically attributed to the combined influence of surface asperity interaction and frictional energy dissipation during tightening. At moderate surface roughness levels, improved asperity interlocking and contact stability promote more effective torque transfer into preload generation. However, excessively high roughness and friction conditions increase localized sliding resistance and frictional energy losses, thereby reducing the efficiency of torque-to-preload conversion and decreasing the effective nut factor (K).

***Effect of (HB) and (Ra) on (K):*** Fig 9.b illustrates that (K) significantly decreases with increasing material hardness, particularly at high roughness values. The interaction between surface roughness and material hardness is associated with the deformation behavior of surface asperities under compressive loading. Softer materials are more susceptible to localized plastic deformation and indentation at rough contact interfaces, which alters the real contact area and contact pressure distribution during tightening. In contrast, harder materials exhibit reduced asperity deformation and more stable load transfer characteristics, leading to lower effective friction and reduced nut factor values.

***Effect of (HB) and (μ) on (K):*** Fig 9.c reveals that (K) reaches relatively high values under conditions of low material hardness and moderate-to-high friction coefficient. At high hardness and low friction levels, (K) decreases noticeably due to reduced contact interaction and lower frictional contribution to preload transfer. This interaction indicates that frictional conditions and material deformation characteristics jointly govern the torque-to-preload conversion efficiency in bolted joints.

Overall, the nut factor K is influenced by nonlinear effects and strong interactions between input factors. Therefore, a second-order regression model is necessary to accurately predict K and determine optimal design conditions for bolted joints.

## Reliability-Based Design Optimization (RBDO)

### Probability distribution models of input parameters

In practical manufacturing and assembly processes, key design variables such as the composite friction coefficient (μ), surface roughness (Ra), and material hardness (HB) inherently exhibit random variability due to machining errors, lubrication inconsistencies, and material heterogeneity. To realistically represent operating conditions and ensure design reliability, these parameters were modeled as independent Gaussian random variables.

Although surface roughness may indirectly influence the friction coefficient through contact behavior, the investigated variables were treated as statistically independent in the present study due to the controlled experimental design and parameter selection procedure. Specifically, the friction coefficient was determined from measured torque–preload relationships under controlled tightening conditions, whereas surface roughness and material hardness were independently prescribed through machining and material selection processes. In addition, no significant linear dependency among the investigated variables was observed within the experimental dataset. Therefore, the independence assumption was considered acceptable for the probabilistic modeling and subsequent RBDO analysis.

Specifically, the friction coefficient (μ) was calculated from experimentally measured tightening torque and axial preload, with an estimated standard deviation of σ_μ_ = 0.0167. Surface roughness (Ra), measured using a contact profilometer in accordance with ISO 1997, was characterized by a standard deviation of σ_Ra_ = 0.0167 μm. Material hardness (HB), determined through Brinell hardness testing following ISO 6506−1, exhibited a standard deviation of σ_HB_ = 3.33.

The normality assumption of the stochastic input variables was statistically verified using the Shapiro–Wilk normality test. For all investigated variables, the obtained (p)-values were greater than 0.05, indicating that the null hypothesis of normal distribution could not be rejected at the 95% confidence level. Therefore, the Gaussian distribution assumption was considered statistically appropriate for the subsequent reliability analysis and RBDO procedure.

The adoption of Gaussian distributions is also consistent with common reliability engineering practice for manufacturing-related parameters exhibiting random and unbiased deviations around nominal values. Since the investigated variables are influenced by cumulative effects of machining tolerances, lubrication variability, surface finishing conditions, and material heterogeneity, the normal distribution provides a reasonable approximation for describing their stochastic behavior within the investigated operating range. Furthermore, normally distributed variables facilitate efficient implementation of Monte Carlo simulation and reliability-based design optimization (RBDO). These values are summarized in Table 5.

**Table 5:** Manufacturing tolerances and standard deviations.

Design Variables	Tolerances	Standard deviation (σ)	Probability distribution
Combined friction coefficient (μ)	±0.05	0.0167	Normal $\left(\overset{―}{\mu },\sigma_{\mu }\right)$
Surface roughness (Ra), μm	±0.05	0.0167	Normal $\left(\overset{―}{R}a,\sigma_{Ra}\right)$
Material hardness (HB), HB	±10	3.33	Normal $\left(\overset{―}{H}\overset{―}{B},\sigma_{HB}\right)$

The three input parameters, including friction coefficient (μ), surface roughness (Ra), and material hardness (HB), were selected because of their significant influence on load distribution behavior in threaded joints and their practical relevance to manufacturing and assembly conditions. The friction coefficient directly governs the conversion efficiency between tightening torque and clamping force, varying from 0.1 to 0.3 depending on lubrication and surface conditions. Surface roughness affects the actual contact area, frictional behavior, and preload stability; therefore, roughness levels of Ra = 1.6 μm and 6.3 μm were investigated. Material hardness influences the load-sharing capability between the bolt and clamped members. Accordingly, hardness levels of approximately HB ≈ 120 and 250 were selected to represent materials ranging from aluminum alloys to hardened steels, enabling quantitative assessment of their influence on the nut factor (K).

### Limit state functions of the nut factor

In mechanical design problems with reliability considerations, the limit state function defines the boundary between safe operation and failure. The selection of the nut factor range (0.2 < K < 0.3) is based on experimental observations, engineering practice, and reliability requirements for torque-controlled bolted joints. Standards such as VDI 2230 and International Organization for Standardization 16047 indicate that tightening behavior and preload generation are strongly influenced by friction conditions, lubrication, surface roughness, and material properties. Previous experimental studies have also shown that the nut factor (K) commonly falls within an approximate range of 0.1–0.3 under practical assembly conditions. Among these values, the interval (0.2 < K < 0.3) is generally regarded as a stable operating region that provides sufficient preload while reducing the risks of excessive tightening, preload scatter, and self-loosening during service.

Furthermore, in the context of Reliability-Based Design Optimization (RBDO), this interval facilitates the formulation of probabilistic constraints to ensure that the bolted joint satisfies the required reliability level with high confidence. Therefore, the limit state functions were established based on the allowable safety criterion (0.2 < K < 0.3).

The two limit state functions are constructed as follows:


$$\begin{array}{c} \text{g1}(\text{X})\;=\text{ g1}(\mu ,\text{Ra},\text{HB})\;=\;{\text{K}}_{\text{max}}\;-\text{ K}(\mu ,\text{Ra},\text{HB})\;=\;\text{0}.\text{3}\;-\text{ K}(\mu ,\text{Ra},\text{HB}) \end{array}$$ (11)

$$\begin{array}{c} \text{g2}(\text{X})\;=\text{ g2}(\mu ,\text{Ra},\text{HB})\;=\text{ K}(\mu ,\text{Ra},\text{HB})\;-\;{\text{K}}_{\text{min}}\;=\text{ K}(\mu ,\text{Ra},\text{HB})\;-\;\text{0}.\text{2} \end{array}$$ (12)

where, K(μ,Ra,HB) is calculated using a second-order regression model derived from experimental data.

A design is considered safe when both conditions g_1_(X) > 0 and g_2_(X) > 0 are simultaneously satisfied. This model is used to evaluate the probability of satisfying the design requirements in reliability analysis and RBDO. The limit state functions are applied to assess system reliability and guide optimal design under random variability in input parameters.

### RBDO formulation and solution

#### RBDO formulation.

The RBDO problem is formulated to find the optimal combination of assembly parameters (μ,Ra,HB) such that the nut factor K achieves the lowest possible mean value while still ensuring that the probability of operating within the safe range exceeds 95%. In this study, the objective function is defined as the expected value of the nut factor K, aiming to reduce the average load transferred to the bolt during service. The optimization model is formulated as follows:

Objective function:


$$\begin{array}{c} \underset{\mu ,Ra,HB}{min}\begin{array}{cc} & E\left[K\left(\mu ,Ra,HB\right)\right] \end{array} \end{array}$$ (13)

where, K(μ,Ra,HB) represents a second-order regression model derived from experimental data

Subject to:

Constraints on the design domains of the input variables:

0.1 ≤ μ ≤ 0.3
1.6 ≤ Ra ≤ 6.3
120 ≤ HB ≤ 250

Reliability constraint: Guarantee the probability that the nut factor remains within the allowable safety range


$$\begin{array}{c} \text{P}(\text{0}.\text{2}\;<\text{ K}(\mu ,\text{Ra},\text{HB})\;<\;\text{0}.\text{3})\;\geq \;\text{0}.\text{999} \end{array}$$ (14)

To summarize, the reliability-based design optimization (RBDO) problem for the bolted joint can be expressed as follows:


$$\begin{array}{c} \text{Designvariables:\textbackslash{}hspace\{0.33em}\}\mu ,Ra,HB \end{array}$$

$$\begin{array}{c} \text{Objectivefunction:\textbackslash{}hspace\{0.33em}\}\underset{\mu ,Ra,HB}{min}\begin{array}{cc} & E\left[K\left(\mu ,Ra,HB\right)\right] \end{array} \end{array}$$ (15)

Subject to:


$$\begin{array}{c} \left\{ \begin{array}{c} @c@0.1\leq \mu \leq 0.3\\ 1.6\leq Ra\leq 6.3\\ 120\leq HB\leq 250\\ \text{P(0.2}<K(\mu \text{,Ra,HB})<0.3)\geq \text{0.999} \end{array}\right. \end{array}$$

Minimizing the expected nut factor K implies improving the efficiency of torque-to-preload conversion. A lower K value indicates that a larger portion of the applied tightening torque is effectively transformed into axial preload rather than being dissipated by frictional losses. This contributes to more predictable preload levels, reduced scatter in clamping force, and improved joint reliability under torque-controlled tightening.

#### Solving the design optimization problem.

The Monte Carlo method combined with the Genetic Algorithm (GA) is employed to solve the Reliability-Based Design Optimization (RBDO) problem for the nut factor model (K). This approach enables direct handling of the randomness in the input variables as well as the probabilistic constraint function, without requiring gradient information.

For the optimization process, the Genetic Algorithm (GA) was implemented with 100 candidate solutions in each generation and a maximum iteration limit of 200 generations. Recombination between parent solutions was performed using a single-point crossover strategy with a crossover probability of 0.8, while random perturbations were introduced through a uniform mutation operator with a mutation probability of 0.05. To preserve the best solutions during the evolutionary process, elitism was applied by retaining the highest-performing individuals in each generation.

The optimization procedure was considered converged when the best objective value showed no noticeable improvement over 25 successive generations, or when the predefined maximum number of generations was reached. In order to improve reproducibility and consistency of the optimization results, a fixed random seed (seed = 2500) was assigned throughout all simulations.

Furthermore, the GA optimization was repeated independently 30 times to evaluate the robustness and repeatability of the obtained solutions. The statistical variation of the optimal fitness values and design variables was then analyzed using their corresponding mean and standard deviation.

**Fig 10:**
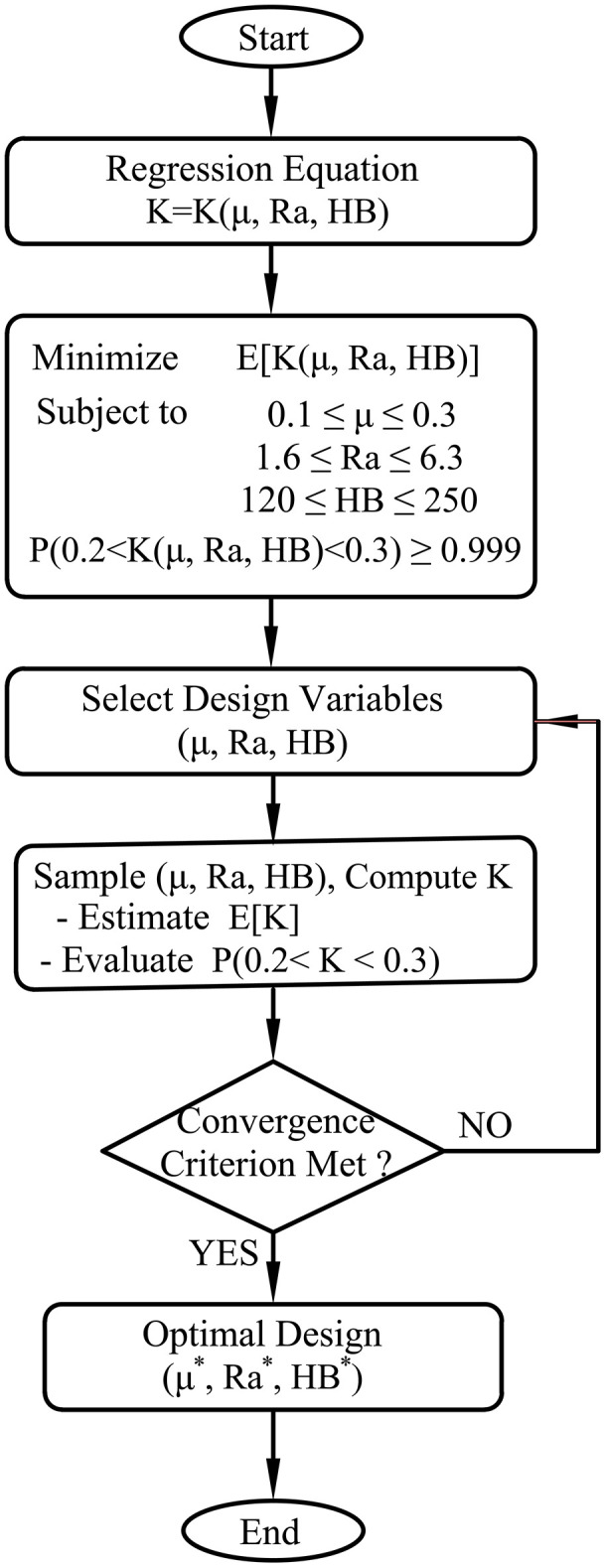
Overall procedure for solving the RBDO problem.

The overall procedure for solving the RBDO problem is illustrated in Fig 10.

To solve the nonlinear RBDO problem with probabilistic constraints, the Genetic Algorithm (GA) is employed to find the optimal solution set (μ,Ra,HB). GA is well-suited for handling non-differentiable problems, particularly when the objective function and constraints are evaluated through Monte Carlo simulations, as in the case of the nut factor K, which exhibits a complex probability distribution.

Compared with deterministic optimization approaches such as RSM-based desirability methods, Simulated Annealing (SA), and hybrid GRA–PCA techniques reported in previous studies [31], the present work focuses on a probabilistic Reliability-Based Design Optimization (RBDO) framework in which uncertainty propagation and probabilistic constraints are explicitly considered. In this context, the Genetic Algorithm (GA) provides advantages in handling nonlinear and nonconvex optimization problems involving stochastic variables without requiring gradient information.

Previous studies have demonstrated that regression-based and hybrid optimization models can achieve high predictive capability when combined with appropriate statistical validation procedures. For example, the study reported in [31] employed an RSM-GRA–PCA hybrid optimization framework and obtained strong agreement between predicted and experimental results through confirmatory experiments. Similarly, the regression model developed in the present work achieved a high coefficient of determination R^2^ = 99.95%. However, in addition to the statistical fitting accuracy indicated by R^2^, the proposed framework was further validated through Monte Carlo-based probabilistic analysis and repeated optimization runs to evaluate robustness and consistency under uncertainty.

The results of the RBDO problem are presented in Table 6.

**Table 6:** Optimization results.

Bolted joints		
**Design Variables**	**Value before optimization**	**Value after optimization (RBDO)**
Combined friction coefficient (μ)	[0.1, 0.3]	0.1999
Surface roughness (Ra), μm	[1.6, 6.3]	3.0506
Material hardness (HB), HB	[120, 250]	167.88
**Nut factor (K)**	**[0.2, 0.3]**	**0.216**

## Reliability and sensitivity analysis

### Reliability validation by Monte Carlo simulation

The Monte Carlo Simulation (MCS) method was employed to re-evaluate the reliability of the optimized bolted joint assembly parameters. The reliability results obtained from MCS were compared with the target reliability level (R*), thereby verifying the effectiveness and robustness of the proposed RBDO framework.

**Fig 11:**
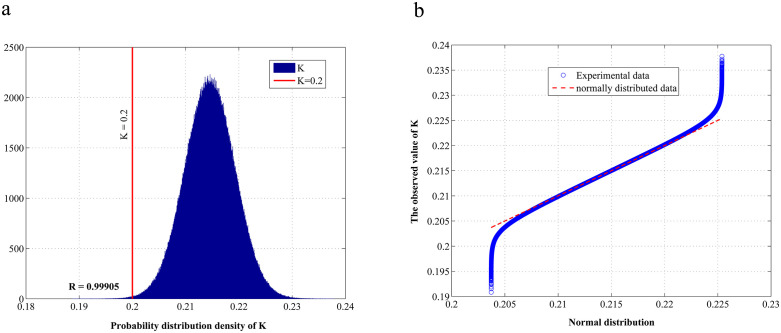
Reliability analysis using Monte Carlo simulation. **(a)** Reliability of K using MCS; **(b)** Q–Q Plot.

To ensure the statistical stability of the reliability estimation, a convergence analysis was performed by progressively increasing the Monte Carlo sample size from 10^4^ to 5 × 10^6^. The estimated reliability value gradually converged and became essentially stable for sample sizes exceeding 10^6^. Therefore, a total of 5 × 10^6^ samples was adopted in this study to provide sufficiently accurate and reliable probability estimation for the target reliability level. The corresponding simulation results obtained with N = 5 × 10^6^ samples are presented in Fig 11.

The reliability analysis result using the Monte Carlo Simulation (MCS) method yields a computed reliability value of R_MCS_ = 0.99905, which is greater than the target value R* = 0.999. This confirms that the bolt joint structure, after being optimized using the RBDO approach, meets the design requirements. The application of Monte Carlo Simulation to re-evaluate the reliability of the optimized design confirms the accuracy and effectiveness of the proposed method.

The Q–Q plot results indicate that the distribution of the nut factor K can be approximated by a normal distribution in the central region; however, noticeable deviations exist in both tails. Therefore, the Monte Carlo method is preferred for accurately estimating the reliability probability within the interval [0.2, 0.3].

### Sensitivity analysis of design variables

To evaluate the influence of each random variable on the system’s reliability, the study applies the One-at-a-Time (OAT) method in combination with Monte Carlo Simulation. By varying one input parameter while keeping the others fixed at their mean values, the change in the probability P(0.2 < K < 0.3) is recorded.

**Fig 12:**
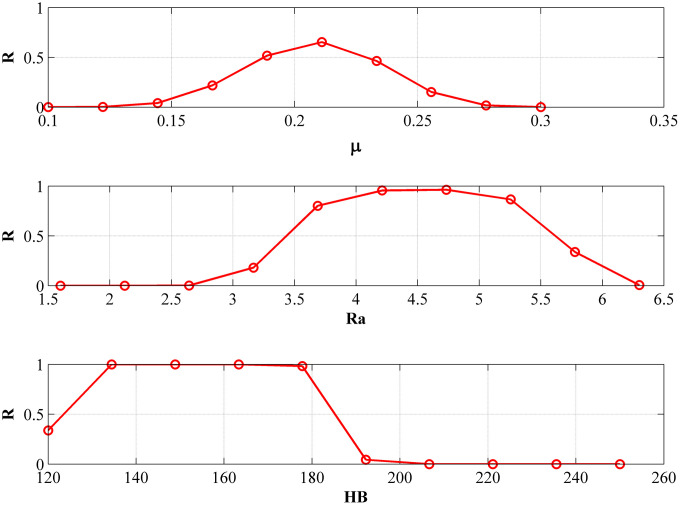
Sensitivity of design variables.

Fig 12 illustrates the sensitivity of the reliability response with respect to each design variable, including the friction coefficient (μ), surface roughness (Ra), and material hardness (HB). The results reveal that the influence of all three variables is nonlinear and dependent on their value ranges. The coefficient of friction (μ) shows the highest sensitivity around μ ≈ 0.22, where small changes significantly affect K.

Although surface roughness (Ra) showed the highest normalized relative influence, the friction coefficient μ exhibited strong local sensitivity in certain operating regions. Therefore, lubrication conditions may significantly influence the robustness of the proposed optimization model. Lubrication can reduce direct asperity interaction and stabilize sliding behavior at the thread and bearing interfaces, thereby decreasing the variability of the friction coefficient and improving preload consistency during tightening. However, different lubrication regimes may also alter the torque–preload relationship through changes in lubricant film thickness and contact mechanics. Therefore, the present optimization results should be interpreted under the dry-contact conditions investigated in this study. Future work should incorporate lubrication conditions as additional stochastic variables in the RBDO framework to evaluate their influence on reliability and optimization robustness more comprehensively

Surface roughness (Ra) has a strong impact in the range of 3 to 5.5 µm, while material hardness (HB) exhibits high sensitivity when HB ranges from 120 to 180. Beyond these regions, the effect of each factor on K becomes minimal. These findings underscore the importance of considering range-specific behavior in design and optimization, and further justify the application of second-order response surface modeling and reliability-based design optimization (RBDO) in this study.

The nonlinear influence of surface roughness on the nut factor K can be explained by the combined effects of real contact area and asperity interaction at the mating surfaces. For very smooth surfaces, the real contact area becomes relatively large, promoting stable sliding conditions and lower frictional resistance during tightening. In contrast, highly rough surfaces reduce the effective contact area because contact occurs mainly at isolated asperity peaks. Under tightening pressure, these asperities may undergo local plastic deformation or flattening, partially reducing the resistance to sliding.

At moderate roughness levels (Ra ≈ 3–4 μm), the asperities are sufficiently pronounced to produce stronger mechanical interlocking while still maintaining significant surface contact. This condition increases frictional energy dissipation during tightening, resulting in higher torque losses and consequently larger nut factor K values. The observed nonlinear behavior therefore reflects the competing effects between contact area enlargement and asperity interlocking mechanisms

### Influence via standard deviation

The standard deviation represents the extent of random variability in each input variable within the RBDO model. When random variables such as the friction coefficient (μ), surface roughness (Ra), and material hardness (HB) exhibit large standard deviations, the propagated uncertainty through the regression model affects the reliability of the output specifically, the nut factor K.

To assess the influence of each variable, each parameter is perturbed by one standard deviation from its mean value, while the remaining parameters are kept constant. This analysis is performed using Monte Carlo simulation with a large number of samples to compute the probability that K falls within the safe interval 0.2 < K < 0.3. The corresponding change in reliability R for each variable is recorded as ΔR.

The relative impact is normalized using the formula:


$$\begin{array}{c} I_{i}=\frac{\left|\Delta R_{i}\right|}{\sigma_{i}} \end{array}$$ (16)

In addition to conventional sensitivity analysis, this study evaluates the relative influence of random variables by normalizing according to their standard deviations. The results show that the friction coefficient (μ), despite having a small standard deviation, causes a significant change in reliability, leading to the highest relative influence. The material hardness (HB) has a large absolute impact, but due to its high standard deviation, its relative influence is lower. Meanwhile, the surface roughness (Ra) has a very minor effect, both in absolute and relative terms. The results of relative influence of the random variables are presented in Table 7.

**Table 7:** Relative influence of the random variables.

Random variables	Standard deviation σ_i_	Variation ΔR_i_	Relative influence I_i_ = ΔR_i_/σ_i_
Combined friction coefficient (μ)	0.0167	0.013	0.7798
Surface roughness (Ra), μm	0.0167	0.0204	1.2237
Material hardness, HB	3.33	0.3498	0.1049

The results indicate that the Surface roughness (Ra) exhibits the highest relative influence, as even a small change in its mean value leads to a significant variation in reliability. In contrast, material hardness (HB) has a greater absolute impact, but due to its larger standard deviation, its relative influence is reduced. Although the friction coefficient (μ) exhibits strong local sensitivity in certain operating regions, its normalized relative influence is lower than that of surface roughness (Ra). This analysis provides a clear prioritization for controlling input variables in reliability-based design optimization.

**Fig 13:**
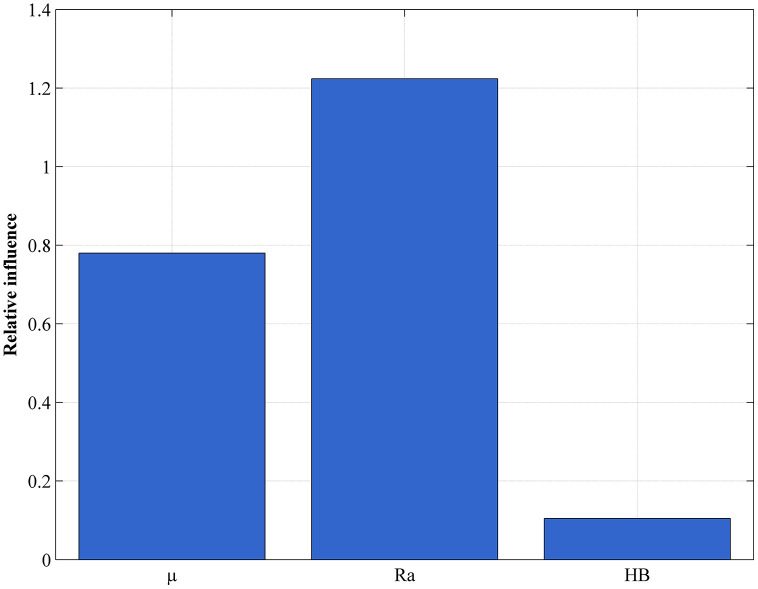
Relative influence of the random variables.

Fig 13 presents a bar chart comparing the relative influence levels of the random variables. The results show that the surface roughness (Ra) is the most influential variable on system reliability, while the friction coefficient (μ) has the least effect. Standard deviation-based normalization ensures a fair assessment of each variable’s sensitivity, supporting the prioritization of design variables in reliability-based optimization.

## Comparison of optimization approaches

**Fig 14:**
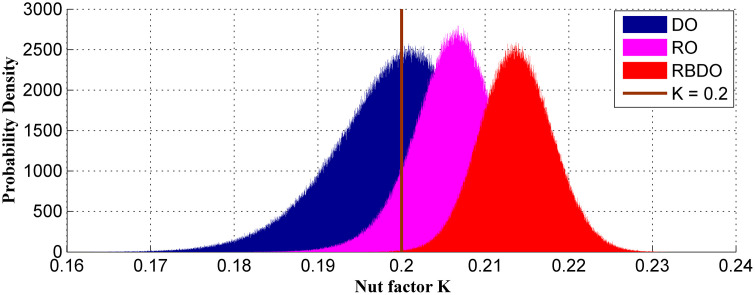
Comparison of results among three optimal design methods.

Table 8 and Fig 14 present a comparison among three optimization strategies: Deterministic Optimization (DO), Robust Optimization (RO), and Reliability-Based Design Optimization (RBDO). The comparative results clearly demonstrate the advantages of the probabilistic RBDO framework under uncertain operating conditions.

**Table 8:** Comparison of optimal solutions obtained from DO, RO, and RBDO.

optimal design methods	Optimal Solution (μ,Ra,HB)	Expectation E[K]	Probability P(0.2 < K < 0.3)	Remarks
DO	(0.2618, 5.47, 175.83)	0.2	0.4912	Non-compliance
RO	(0.1815, 2.88, 170.49)	0.208	0.89620	Stable results
RBDO	(0.1999, 3.05, 167.88)	0.216	0.99905	Highest reliability performance

Although the optimal solution obtained from DO yields the minimum expected value of the nut factor (E[K]=0.2), the probability of satisfying the safety condition is only 49.12%, indicating insufficient reliability in practical assembly applications. This result highlights the limitation of deterministic optimization methods, which optimize nominal performance without explicitly accounting for uncertainty propagation.

In contrast, the RO-based design improves the stability of the output response and achieves a higher probability of satisfying the safety requirement (89.62%). However, due to the absence of explicit probabilistic constraints, the RO solution still carries a non-negligible risk of violating the allowable safety range.

The proposed RBDO approach incorporates probabilistic constraints directly into the optimization process. As a result, the optimized solution provides a safety satisfaction probability of 99.905%, exceeding the target reliability level (≥99.9%). Although the expected value of the nut factor slightly increases to (E[K]=0.216), the obtained design exhibits significantly improved reliability and robustness under uncertainty.

In addition to the RBDO method, the study also investigates the Robust Optimization (RO) approach, which aims to minimize the variability of the nut factor K under assembly noise. The results show that the RO-based design achieves an expected value of K = 0.208, and the output distribution is more concentrated and stable compared to DO. However, due to the absence of an explicit probability constraint, RO still carries a small risk (≈10.38%) of violating the safety condition. In contrast, the distribution from the RBDO method is centered around the mean value K = 0.216, which is concentrated within the allowable range and ensures a safety satisfaction probability of 99.905%.

The results demonstrate that the RBDO is a more reliable optimization approach for design problems subjected to uncertainty. Although it involves greater computational effort, RBDO ensures a high probability of constraint satisfaction, thereby demonstrating its potential applicability for reliability-oriented design problems in mechanical engineering, aerospace, and structural engineering under uncertainty.

To validate the proposed RBDO framework, additional confirmation tightening experiments were conducted at the optimized parameter combination (μ = 0.1999, Ra = 3.05 μm, HB = 167.88). The experimentally measured nut factor was approximately (K = 0.232), while the predicted value obtained from the probabilistic optimization model was (E[K] = 0.216). The relative deviation between the experimental and predicted results remained within an acceptable range, demonstrating good agreement between the experimental measurements and the proposed regression-based RBDO framework.

To further investigate the influence of reliability constraints on the location of the optimal solution, additional RBDO analyses were performed under different target reliability levels. The corresponding results are summarized in Table 9.

**Table 9:** Sensitivity of optimal solutions under different reliability constraints.

Reliability constraint	Optimal solution (μ, Ra, HB)	E[K]	P(0.2 < K < 0.3)	Observation
≥ 0.990	(0.2281, 3.8573, 170.04)	0.218	0.99070	Solution located closer to the design boundary
≥ 0.995	(0.2142, 3.5896, 173.76)	0.213	0.99509	Intermediate feasible solution
≥ 0.999	(0.1999, 3.0500, 167.88)	0.216	0.99905	Conservative interior solution

The results indicate that the optimal solution gradually shifts toward the interior region of the design space as the required reliability level increases. Under less stringent probabilistic constraints, the optimization tends to approach boundary regions to achieve lower objective values more aggressively. In contrast, stricter reliability requirements promote more conservative parameter combinations with reduced variability and improved probability of constraint satisfaction.

The non-monotonic variation of some design variables is attributed to the nonlinear interactions among the input parameters and the trade-off between minimizing the expected nut factor and satisfying the probabilistic reliability constraint. Therefore, the optimal solution is not expected to vary monotonically with the imposed reliability level.

Nevertheless, it should be noted that the present study primarily focuses on the tightening-stage reliability behavior of bolted joints through the analysis of friction conditions, surface roughness, and material hardness. Time-dependent effects such as preload relaxation, embedding of contact surfaces, and cyclic loading were not explicitly considered in the current RBDO formulation.

In practical applications, these factors may significantly influence preload retention and long-term joint reliability. In particular, preload relaxation and embedding effects can reduce the effective clamping force over time, while cyclic loading may accelerate self-loosening and fatigue-related degradation.

Moreover, real assembly environments may introduce additional sources of uncertainty associated with lubrication variability, operator-dependent tightening procedures, and tightening tool control accuracy. In the present study, the stochastic behavior of the combined friction coefficient partially reflects the influence of varying lubrication conditions during assembly. However, human-related factors such as operator skill, tightening sequence, tightening speed, and real-time tool deviations were not explicitly incorporated into the current probabilistic framework. These factors may further affect preload consistency and long-term assembly reliability in industrial applications.

Therefore, incorporating preload loss models, cyclic loading conditions, and practical assembly-process uncertainties into the probabilistic reliability framework represents an important direction for future research.

## Conclusions

This study experimentally and statistically investigated the influence of the friction coefficient (μ), surface roughness (Ra), and material hardness (HB) on the nut factor (K) in bolted joints. Using a Box–Behnken experimental design with 15 test runs, a second-order regression model was established, demonstrating strong predictive performance with an R² value of 99.95% and no significant lack of fit (p = 0.266).

The regression results indicate that the friction coefficient and material hardness are the dominant factors affecting the nut factor, while surface roughness also exerts a significant influence, particularly through interaction effects and reliability sensitivity. Experimentally, increasing the friction coefficient from 0.1 to 0.3 resulted in an increase of K from approximately 0.12 to 0.30 under low-hardness conditions (HB ≈ 120). In contrast, increasing material hardness from HB ≈ 120 to HB ≈ 250 reduced the nut factor by up to 40%, reflecting improved load distribution and reduced contact deformation. Surface roughness showed a nonlinear effect, with moderate roughness levels (Ra ≈ 3–4 μm) leading to higher K values compared to very smooth or rough surfaces.

Based on the experimentally derived regression model, a reliability-based design optimization (RBDO) framework was implemented using Monte Carlo simulation and a genetic algorithm. The optimal design was obtained at μ = 0.1999, Ra = 3.05 μm, and HB = 167.88, yielding an expected nut factor of E[K] = 0.216 while satisfying the probabilistic constraint P(0.2 < K < 0.3) ≥ 0.999. A subsequent Monte Carlo validation with 5 × 10⁶ samples confirmed a reliability level of 0.99905, exceeding the prescribed target.

Overall, the primary contribution of this study lies in the quantitative identification of the relationships among key assembly parameters and the nut factor, supported by statistically significant regression coefficients and interaction terms obtained from the Box–Behnken methodology. The proposed probabilistic framework may provide a useful foundation for future reliability-oriented bolted joint design studies under uncertain assembly conditions.

The investigated hardness range (120–250 HB) mainly represents conventional structural steels. Materials used in aerospace or lightweight applications, such as aluminum alloys or ultra-high-strength steels, may exhibit different elastic-plastic contact behavior and torque–preload characteristics. Therefore, the regression model developed in this study should be interpreted within the investigated material range only. Nevertheless, the proposed experimental and RBDO framework may be extended to other material systems through appropriate experimental recalibration and validation.

Although the present study was conducted specifically on M14 bolted joints, the authors recognize that the nut factor (K) is also influenced by geometric parameters such as nominal diameter, thread pitch, and thread profile. Variations in bolt size may alter the contact area, thread friction behavior, stiffness distribution, and torque-to-preload conversion efficiency. Therefore, the regression coefficients obtained in this work are specific to the M14 configuration and should not be directly extrapolated to other fastener sizes without additional experimental validation.

Nevertheless, the proposed framework combining Box–Behnken experimental design, regression modeling, Monte Carlo simulation, and Reliability-Based Design Optimization (RBDO) remains general and transferable to other bolted joint geometries. Future studies should extend the methodology to different bolt sizes (e.g., M10, M12, and M16) in order to establish generalized predictive models and investigate the influence of geometric scaling on the nut factor and reliability behavior.

### Future research directions

Future studies may extend the proposed probabilistic framework by incorporating additional assembly and geometric factors such as lubrication conditions, surface coatings, thread geometry, tightening speed, washer configuration, and tightening tool variability. These parameters may introduce coupled nonlinear interactions and additional uncertainty sources affecting the torque–preload relationship and the variability of the nut factor under practical assembly conditions.

Moreover, time-dependent phenomena including preload relaxation, embedding effects, cyclic loading, and vibration should be further investigated to better characterize long-term bolted joint reliability under real service conditions. Human-related factors such as operator skill, tightening sequence, and real-time tool control deviations may also be incorporated into future probabilistic assembly models.

From a reliability perspective, future work may consider non-Gaussian probabilistic models, correlated uncertainties, and global sensitivity analysis methods to better capture complex stochastic behaviors in bolted joint systems. In addition, advanced intelligent predictive approaches such as ANFIS-based surrogate models, hybrid machine-learning frameworks, and metaheuristic optimization strategies may be explored to further improve nonlinear prediction capability and computational efficiency in reliability-based optimization.

Finally, future research may focus on multi-objective RBDO formulations that simultaneously consider the mean and variance of the nut factor (K), preload consistency, tightening scatter, fatigue performance, and long-term reliability under uncertainty.

## Supporting information

S1 File Experimental data for the regression Equation (10). (RAR)

## References

[1] Loc NH. Fundamental of machine design: National University Publisher HCMC (in Vietnamese); 2016.

[2] Tran VT, Nguyen HL. Experimental investigation of the influence of friction, surface roughness and material hardness on the external load factor in threaded joints. PLoS One. 2026;21(1):e0340073. doi: 10.1371/journal.pone.0340073 41490080 PMC12768235

[3] Budynas RG, Nisbett JK. Shigley’s mechanical engineering design. New York: McGraw-Hill. 2011.

[4] Alsardia T, Lovas L. Investigation of the effect of the surface treatment and lubrication during repeated tightening on the nut coefficient of a bolted joint using the Taguchi method. Jordan Journal of Mechanical & Industrial Engineering. 2024;18(1).

[5] Chen Y, Yamaguchi T, Hayashi G, Yamauchi M, Ueno K. Experimental study on long friction-type bolted joint combined with interference fit bolt. Advances in Bridge Engineering. 2024;5(1):6.

[6] He T, Chen W, Liu Z, Gong Z, Du S, Zhang Y. The impact of surface roughness on the friction and wear performance of GCr15 bearing steel. Lubricants. 2025;13(4):187. doi: 10.3390/lubricants13040187

[7] Stang N. Effect of surface roughness and external loading on embedment in steel and aluminum bolted joints. South Dakota State University. 2021.

[8] Jalali H, Khodaparast HH, Friswell MI. The effect of preload and surface roughness quality on linear joint model parameters. Journal of Sound and Vibration. 2019;447:186–204. doi: 10.1016/j.jsv.2019.01.050

[9] Wettstein A, Matthiesen S. Investigation of the thread coefficient of friction when impact tightening bolted joints. Forsch Ingenieurwes. 2019;84(1):55–63. doi: 10.1007/s10010-019-00392-z

[10] Lee J, Kim D, Seok CS. Methodology for evaluating the tightening torque-clamping force relationship and friction coefficients in bolted joints. J Mech Sci Technol. 2022;36(4):1913–9.

[11] Yan X, Liu Z, Zheng M, Li Y, Wang Y, Chen W. Preload control method of threaded fasteners: A review. Chin J Mech Eng. 2024;37(1):97.

[12] Liu Z, Zheng M, Yan X, Zhao Y, Cheng Q, Yang C. Changing behavior of friction coefficient for high strength bolts during repeated tightening. Tribology International. 2020;151:106486. doi: 10.1016/j.triboint.2020.106486

[13] Wang C, Sun Q, Han S. Effect of surface parameters on anti-loosening performance of bolts for engineering machinery. Matéria (Rio J). 2023;28(3). doi: 10.1590/1517-7076-rmat-2023-0159

[14] Li Z, Chen Y, Sun W, Jiang P, Pan J, Guan Z. Study on self-loosening mechanism of bolted joint under rotational vibration. Tribology International. 2021;161:107074. doi: 10.1016/j.triboint.2021.107074

[15] Li Y, Lang L. Theoretical and experimental study on the damage behavior of bolted joints under cyclic loads. Elsevier. 2024.

[16] Qiu PQ, Wang WW, Wang K, Zhang XQ, Ning JG, Zhao CL. Experimental study on the energy dissipation mechanism of bolted rock under dynamic loading. Sci Rep. 2025;15(1):17182. 40382447 10.1038/s41598-025-02436-7 PMC12085676

[17] Yu Q, Yang X, Zhou H. An experimental study on the relationship between torque and preload of threaded connections. Adv Mech Eng. 2018;10(8):1687814018797033.

[18] Montgomery DC. Design and analysis of experiments. John Wiley & Sons. 2017.

[19] Gunasekaran J, Sevvel P, Solomon IJ, Roy JV. Optimization of FSW parameters using SA Algorithm and ANFIS-Based Models to Maximize Mechanical Properties of AZ80A Mg Alloy Joints. J of Materi Eng and Perform. 2024;34(14):14487–506. doi: 10.1007/s11665-024-10062-z

[20] Chen Y, Huang X, Ma M, Luo J, Ding B. Reliability-based design optimization of key components in a gantry machining center. Probabilistic Engineering Mechanics. 2025;81:103786. doi: 10.1016/j.probengmech.2025.103786

[21] Gholami P, Esmaeili Yengejeh S, Farsi MA. Reliability-based design optimization of composite pipes subjected to internal hydrostatic pressure fatigue. Composites Communications. 2025;57:102484. doi: 10.1016/j.coco.2025.102484

[22] Chen J, Chen Z, Jiang W, Guo H, Chen L. A reliability-based design optimization strategy using quantile surrogates by improved PC-kriging. Reliability Engineering & System Safety. 2025;253:110491. doi: 10.1016/j.ress.2024.110491

[23] Peng Y, Chang T. Reliability-based design optimization of tuned mass-damper-inerter for vibration mitigation of spar-type floating offshore wind turbines. Ocean Engineering. 2024;310:118630. doi: 10.1016/j.oceaneng.2024.118630

[24] Loc NH, Thuy TV, Trung PQ. Reliability-based analysis of machine structures using second-order reliability method. JAMDSM. 2019;13(3):JAMDSM0063–JAMDSM0063. doi: 10.1299/jamdsm.2019jamdsm0063

[25] Khodaygan S, Sharafi MH. A New Approach for the Reliability-Based Robust Design Optimization of Mechanical Systems under the Uncertain Conditions. SAE Technical Paper Series, 2018. doi: 10.4271/2018-01-0615

[26] Nguyen HL. Reliability based design and analysis of mechanical systems: National University publisher HCMC, Viet Nam; 2015.

[27] Zhao J, Gong X, Yao Z, Zhao C, Xu M, Shi G, et al. Reliability-based design optimization of key components in the ball screw feed system. Mechanism and Machine Theory. 2025;214:106151. doi: 10.1016/j.mechmachtheory.2025.106151

[28] Zhang D, Yang M, Han X. A decoupled method for time-dependent reliability-based design optimization. Sci China Technol Sci. 2024;68(1). doi: 10.1007/s11431-024-2830-5

[29] Hu W, Cheng S, Yan J, Cheng J, Peng X, Cho H, et al. Reliability-based design optimization: A state-of-the-art review of its methodologies, applications, and challenges. Struct Multidisc Optim. 2024;67(9). doi: 10.1007/s00158-024-03884-x

[30] Nguyen HL. Design and analysis of experiments. HCMC, Viet Nam: National University publisher. 2021.

[31] D S, P S, Babu S D D, Roy V. Optimization of parameters and formulation of numerical model employing GRA–PCA and RSM approach for friction stir welded Ti–6Al–4V alloy joints. Mater Res Express. 2024;11(5):056511. doi: 10.1088/2053-1591/ad48e3

